# Polystyrene Nanoplastics Induce Lung Injury via Activating Oxidative Stress: Molecular Insights from Bioinformatics Analysis

**DOI:** 10.3390/nano12193507

**Published:** 2022-10-07

**Authors:** Tianyi Zhang, Sheng Yang, Yiling Ge, Xin Wan, Yuxin Zhu, Jie Li, Lihong Yin, Yuepu Pu, Geyu Liang

**Affiliations:** Key Laboratory of Environmental Medicine Engineering, School of Public Health, Southeast University, Ministry of Education, Nanjing 210009, China

**Keywords:** nanoplastics, lung injury, RNA-sequencing, oxidative stress

## Abstract

(1) Background: Increasing evidence reveals that airborne plastic particles will continue to degrade into nanoplastics which are then inhaled by humans, causing injury to the respiratory system with controversial molecular mechanisms. (2) Methods: We used polystyrene nanoplastics (PS-NPs) as the representative pollutants to explore the inhalation toxicology of nanoplastics and identified the potential mechanism through high-throughput sequencing. (3) Results: PS-NPs inhibited cell viability in a dose-dependent manner and 0 μg/cm^2^, 7.5 μg/cm^2^ and 30 μg/cm^2^ PS-NP-treated groups were selected for RNA-seq. Gene Ontology (GO) and Kyoto Encyclopedia of Genes and Genomes (KEGG) analysis suggested that lung injuries caused by PS-NPs were mediated via redox imbalance, which was verified by reactive oxygen species (ROS) staining. Additionally, we obtained ten key transcription factors (TFs) governing differentially expressed genes (DEGs), nine of which were involved in the regulation of oxidative stress. An oxidative stress-associated TF-mRNA regulatory network was constructed on account of the findings above. Further joint analysis with animal experiment data from the GEO database identified a crucial oxidative stress-related molecule, TNFRSF12A. qRT-PCR was performed to confirm the results of RNA-seq. (4) Conclusions: Our study indicates the potential role of oxidative stress in the mechanism of nanoplastics-induced lung injuries, with several key genes being promising targets to analyze in future investigations.

## 1. Introduction

Plastic materials are widely used all over the world because of their durability, lightness, versatility and flame retardancy [1,2]. It is reported that global plastic output increases rapidly every year and reached 360 million tons in 2020 [3]. Undoubtedly, plastic production and utilization have surpassed most other man-made materials and plastic products have become an integral part of modern lifestyles. However, the inadequate recycling and slow degradation of plastic boost their accumulation in the environment. More worryingly, plastic products are prone to disintegrate into microplastics and nanoplastics gradually through ultraviolet radiation, physical abrasion and biodegradation, some of which will eventually be absorbed into the human body in various manners and cause serious health threats [4,5].

Microplastics are typically considered to have a dimension between 1 μm and 1000 μm, while nanoplastics are considered to be up to 100 nm in size [6]. To date, the impact of micro(nano)plastics on aquatic organisms has long been investigated while their contamination status and eco-toxicological effects in the atmospheric environment remain unclear [7,8,9]. In fact, the adverse influence and potential risks of airborne micro(nano)plastics should not be ignored. Evidence shows that micro(nano)plastics can be released into the air from both indoor and outdoor sources, including clothing fibers, decoration materials, 3D printing, automobile tire erosion, waste incineration and the resuspension of plastic particles in urban dust [10,11,12,13]. Several investigations have quantified the presence of microplastics in the atmosphere of Dongguan (China) [14], Shanghai (China) [15], London (UK) [16] and even in a remote mountain catchment [17]. In addition, microplastics can be detected in human lung tissues [18,19] and isolated from the bronchoalveolar lavage fluid (BALF) of European citizens [20], suggesting that airborne plastic debris could be inhaled and deposited in human alveoli.

Limited by conventional detection technology, nanoplastics are difficult to characterize and most plastic particles reported in the environment are 50 μm to 5 mm in size. But recent research has confirmed that microplastics in the environment will not stop disintegrating into nanoplastics [4,21,22]. Moreover, due to their larger specific surface areas and smaller sizes, nanoplastics are more likely to absorb other chemical substances and possess stronger biological penetration, which may pose a greater threat to the human body. Xu et al. [23] have compared the effects on human lung epithelial cells of polystyrene nanoparticles (PS-NPs) of different sizes (25 nm and 70 nm in diameters). Results show that PS-NPs of smaller sizes are internalized into A549 cells more rapidly and inhibit cell viability at lower concentrations. Gulinare et al. report that PS-NP inhalation increased the risk for pulmonary diseases, such as fibrosis, and PS-NPs with smaller sizes have stronger toxic effects [24]. In addition, targeted metabolomics reveal that PS-NP exposure caused autophagic- and endoplasmic reticulum (ER) stress-related metabolic changes in BEA-2B cells [25]. Therefore, it is of great necessity to attach more attention to respiratory system damage caused by airborne nanoplastics.

Polystyrene, as one of the main components of plastic, has broad applications in electronics, packing products, insulating foams, etc. [26]. Furthermore, a related air sample survey revealed that polystyrene accounts for the greatest fraction of non-fiber microplastics in the London urban region [16]. Moreover, in practical experiments, the higher availability of PS-NPs of various sizes also prompted us to finally choose it as the representative nanoplastic contaminant.

Despite some recent data suggesting a potential association between airborne nanoplastic exposure and pulmonary damage, further research is still needed to fully understand the underlying molecular events. In this study, we performed transcriptome sequencing (RNA-Seq) on human lung epithelial cells (BEAS-2B cells) exposed to low and high concentrations of PS-NPs (40 nm in diameters) to explore the potential mechanisms. We tested two types of human lung epithelial cells, including HPAEpiC and BEAS-2B cells, and then combined our findings with data from animal experiments to identify potential key genes. Our research aimed to offer a fresh viewpoint on the investigation of respiratory toxicity caused by nanoplastics.

## 2. Materials and Methods

### 2.1. Cell Culture and PS-NPs Treatment

BEAS-2B cell line was supplied by the American Type Culture Collection (Manassas, VA, USA) and HPAEpiC cell line was from ScienCell Research Laboratories (San Diego, CA, USA). BEAS-2B cells and HPAEpiC cells were grown in DMEM and an RPMI-1640 medium, respectively, containing 10% fetal bovine serum (Hyclone, Logan, UT, USA) and 1% penicillin-streptomycin antibiotics (Gibco, Waltham, MA, USA). The stock of unmodified polystyrene nanoparticles (PS-NPs) with a size of 40 nm was supplied by Shanghai Huge Biotechnology Corporation (Shanghai, China). The characterization of the PS-NPs was evaluated as previously described [27]. Before exposure to cells, the PS-NPs were treated by ultrasonication to avoid aggregation. The PS-NPs were diluted using a serum-free medium to the corresponding concentrations and exposed to the treated cells.

### 2.2. Cell Viability Assay

Cells were grown in 96-well plates to 70% confluence and then treated with the PS-NP suspension. A total of eleven groups of concentration gradients were set, including 0, 2.5, 5, 7.5, 10, 15, 20, 25, 30, 35 and 40 μg/cm^2^, which were equivalent to 0, 8, 16, 24, 32, 48, 64, 80, 96, 112 and 128 μg/mL with 100 μL culture medium per well in the 96-well plate. After overnight incubation, the CCK-8 assay (Meilune Biotechnology, Dalian, China) was performed to determine cell viability under the guidance of the manufacturer’s protocol.

### 2.3. Measurement of Intracellular Reactive Oxygen Species (ROS) Levels

The intracellular ROS levels were measured using DCFH-DA (Beyotime Biotechnology, Nantong, China) with a fluorescence microscope according to the instructions of the manufacturer. H_2_O_2_ diluted with a serum-free medium to 100 μmol/L was used as the positive control. DCFH-DA was diluted with a serum-free medium to a final concentration of 10 μmol/L. The cell culture medium was then removed, and an appropriate volume of diluted DCFH-DA and H_2_O_2_ was added to fully cover the cells. Cells were incubated at 37 °C for 30 min. Intracellular ROS (DCFH-DA) was detected using fluorescence microscopy with excitation at 488 nm and emission at 525 nm.

### 2.4. RNA-Sequencing

Three concentrations of PS-NP treatment groups (0 μg/cm^2^, 7.5 μg/cm^2^ and 30 μg/cm^2^) were selected for the analysis of the differentially expressed genes (DEGs) and three parallels were set in each group. Nano-Photometer^®^ spectrophotometer (IMPLEN, CA, USA) and Qubit^®^3.0 Fluorometer (Life Technologies, Carlsbad, CA, USA) were used to detect the purity and concentration of the total RNA. The RNA integrity was checked using Agilent 2100 RNA nano 6000 assay kit (Agilent Technologies, Santa Clara, CA, USA). After passing quality control, RNA samples were sequenced using an Illumina system with PE150 as the sequencing strategy. The Fragments per Kilobase per Million Mapped Fragments (FPKM) method was used to calculate the gene expression. The DESeq2 method was used to identify DEGs with the criteria of fold change and FDR (fold change ≥ 2.0 and adjust *p*-value < 0.05). The sequencing depth was 6 Gb per sample.

### 2.5. Bioinformatics Analysis

To better understand the roles of DEGs, a function analysis using the Gene Ontology (GO) and Kyoto Encyclopedia of Genes and Genomes (KEGG) pathways was performed. The GO terminology was described in three parts: biological processes (BPs), cellular components (CCs) and molecular functions (MFs). The KEGG pathway analysis was conducted to enrich the significant signaling pathways.

### 2.6. Prediction of Transcription Factors (TFs) for DEGs and Construction of Oxidative Stress-Associated TF-mRNA Regulatory Network

The DAVID database (http://david.abcc.ncifcrf.gov/, accessed on 8 July 2022) was used to obtain the TFs regulating the DEGs, and 172 TFs were subsequently identified with the criteria of *p*-value < 0.05. After intersecting 172 TFs with the DEGs, we captured ten key TFs that were differentially expressed with the treatment of PS-NPs in BEAS-2B cells. To collect the TFs and DEGs involved in oxidative stress, we searched the keywords “oxidative stress” in the GeneCards database (https://www.genecards.org, accessed on 8 July 2022) to generate the gene set related to oxidative stress, including 9438 candidate genes (Supplementary Table S1) in total. By intersecting the DEGs and TFs with candidate genes, we obtained the genes that were highly related to oxidative stress, which were used to construct the oxidative stress-associated TF-mRNA regulatory network. Cytoscape 3.8.2 software (The Cytoscape Consortium, San Diego, CA, USA) was used to visualize the regulatory network.

### 2.7. Gene Expression of Rat Lung Tissues with Acute Pulmonary Embolism Induced by Polystyrene

The microarray dataset GSE13535 was obtained from Gene Expression Omnibus (GEO, https://www.ncbi.nlm.nih.gov/geo/, accessed on 8 July 2022) which is a public collection of functional genomics data encompassing significant high-throughput sequencing experimental data. The GSE13535 dataset includes a rat lung gene expression during an acute pulmonary embolism. Rats were intravenously injected with 2 concentrations of 25-micron polystyrene (1.3 million polystyrene/100 g weight as low concentration; 2 million polystyrene/100 g weight as high concentration) and 0.15 mL/100 g weight of 0.01% Tween20 as a “vehicle” control. Rats were sacrificed at either 2 h or 18 h after treatment. Finally, we chose the 18 h group for analysis by GEO2R considering that far more DEGs were available in the longer treatment group.

### 2.8. RNA Extraction and Real-Time PCR

A TRIzol (TaKaRa Bio, Shiga, Japan) was used to extract total RNA. Hiscript II Q RT SuperMix (Vazyme, Nanjing, China) was used to perform a reverse transcriptase reaction. Moreover, real-time PCR reactions were performed using SYBR Green PCR Master Mix (Vazyme, Nanjing, China). GAPDH was selected as the internal control and ΔΔCt methods were used for normalization. The Ct values of the target genes were normalized to the control gene, and the mRNA expression was presented as the relative fold change. qRT-PCR experiments were performed in triplicates. The sequences of all the primers for qPCR used in the study are listed in Supplementary Table S2.

### 2.9. Statistical Analysis

Graphpad prism 9.0 (Dotmatics, San Diego, CA, USA) was used for statistical analysis. The experiments were conducted in triplicate and repeated 3 times independently. All the data were expressed as the mean ± standard deviation (SD). A one-way analysis of variance (ANOVA) was performed for statistical difference analysis in the study. *p*-value < 0.05 was considered statistically significant.

## 3. Results

### 3.1. Cytotoxicity of PS-NPs

**Figure 1:**
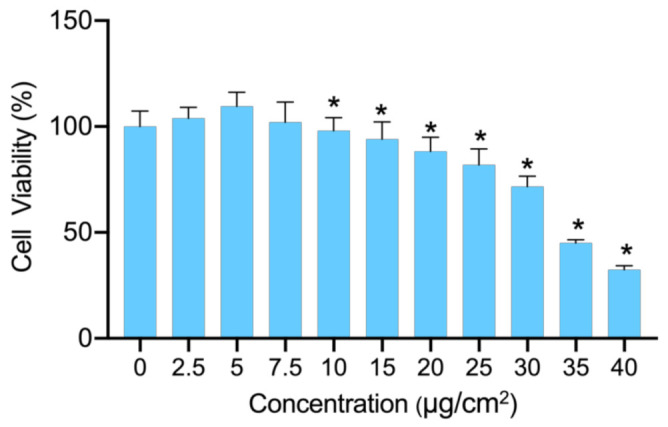
Cytotoxicity of PS-NPs. The viability of BEAS-2B cells after being treated for 24 h by PS-NPs at concentrations ranging from 0 to 40 μg/cm^2^ (*n* = 6). * *p*-value <  0.05 indicates statistically significant differences from the control.

BEAS-2B cells were treated with different concentrations of PS-NPs for 24 h and then the cell viability was measured by CCK-8 assay. As illustrated in Figure 1, the dose-dependent inhibition of cell viability was observed when BEAS-2B cells were exposed to 10–40 μg/cm^2^ PS-NPs. It also revealed that BEAS-2B cells were susceptible to the damage caused by PS-NPs, even at a concentration as low as 10 μg/cm^2^. In addition, the cell viability dropped to approximately 70% when the treated concentration reached 30 μg/cm^2^. For the purpose of capturing the responsive genes induced by PS-NPs in BEAS-2B cells at low-concentration treatment and in a relatively high-concentration group, we selected 7.5 μg/cm^2^ (low-dose group) and 30 μg/cm^2^ (high-dose group) as exposure concentrations and 0 μg/cm^2^ as the control group for the subsequent RNA-seq.

### 3.2. Gene Expression Alternation in PS-NP-treated BEAS-2B Cells

**Figure 2:**
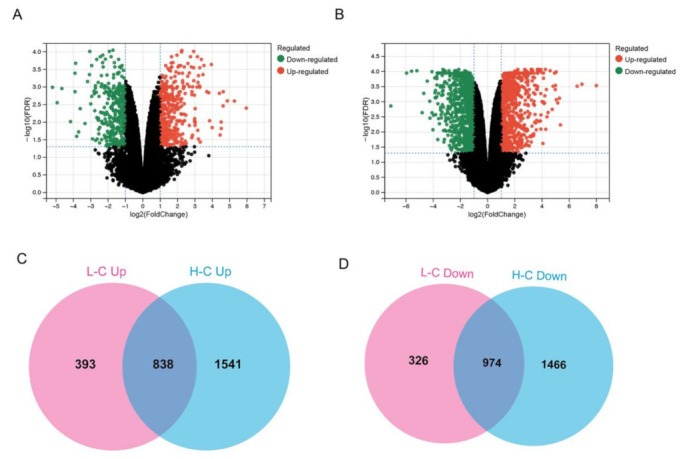
Gene expression alternation in PS-NP-treated BEAS-2B cells. (**A**,**B**): Volcano plots of significantly changed genes after PS-NP exposure at diverse concentrations (**A**) low-dose group vs control group and (**B**) high-dose group vs control group). (**C**,**D**): Up-regulated genes (**C**) and down-regulated genes (**D**) with consistent trend in two concentrations of PS-NP-treated groups. “L-C” denotes low-dose group vs control group, and “H-C” denotes high-dose group vs control group.

We performed the analysis of DEGs in the 0 μg/cm^2^ (control group), 7.5 μg/cm^2^ (low-dose group) and 30 μg/cm^2^ (high-dose group) groups. Compared with the control group, 2531 genes were significantly altered in the 7.5 μg/cm^2^ group, with 1231 genes up-regulated and 1300 genes down-regulated (Figure 2A and Supplementary Figure S1A ). Unsurprisingly, we observed a far more significant number of genetic changes in the 30 μg/cm^2^ group. A total of 4819 genes were remarkably changed in the relatively high-dose group, with 2379 genes up-regulated and 2440 genes down-regulated (Figure 2B and Supplementary Figure S1B). Notably, 1812 genes were chosen for further functional annotation since they showed a consistent trend in both treatment groups compared to the control group, with 838 genes up-regulated (Figure 2C) and 974 down-regulated (Figure 2D) simultaneously.

### 3.3. Functional Annotation of DEGs Induced by PS-NPs and Validation of ROS

**Figure 3:**
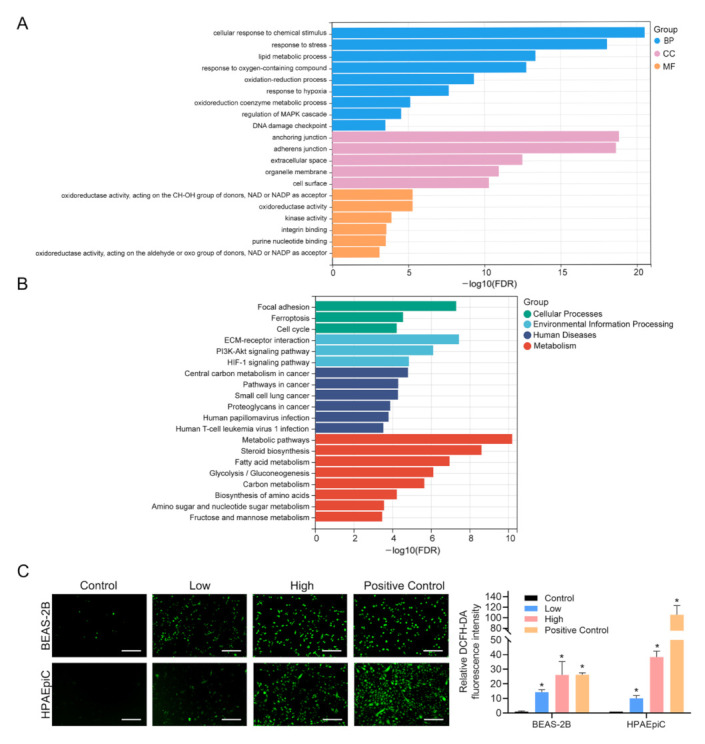
Functional annotation of DEGs induced by PS-NPs and validation of ROS. (**A**,**B**): DEGs with consistent trend in two concentrations of PS-NP-treated groups were selected for GO analysis (**A**) and KEGG pathway analysis (**B**). (**C**) The levels of ROS were detected by DCFH-DA staining assays. Scale bars: 200 μm. ”BP” denotes “Biological Process”, ”CC” denotes “Cell Component” and “MP” denotes “Molecular Function”. * *p*-value <  0.05 indicates statistically significant differences from the control.

In order to achieve the gene function categories and seek the latent pathways of PS-NPs-induced cardiotoxicity, we carried out GO analysis and KEGG pathway analysis based on the DEGs. As illustrated in Figure 3A, we listed 20 remarkable GO terms, which included Cellular response to chemical stimulus, Response to stress, Anchoring junction, Adherences junction, Oxidation-reduction process, Oxidoreductase activity, etc. Notably, approximately 1/4 of the enriched GO terms were closely associated with oxidative stress. Subsequently, we further performed a KEGG pathway enrichment analysis. As illustrated in Figure 3B, we also displayed 20 significant KEGG pathways annotated and marked out as Cellular Processes, Environmental Information Processing, Human Diseases or Metabolism. Similar to GO analysis, we found many KEGG pathways were related to oxidative stress as well, such as ferroptosis, the PI3K-Akt signaling pathway, the HIF-1 signaling pathway, etc. Based on the above results, GO analysis and KEGG pathway analysis implied that PS-NP exposure could spark oxidative stress in BEAS-2B cells. We then performed DCFH-DA staining assays to verify that PS-NPs significantly increased ROS production in BEAS-2B and HPAEpiC cells after exposure for 24 h (Figure 3C).

### 3.4. Prediction of TFs for DEGs and Construction of Oxidative Stress-Associated TF-mRNA Regulatory Network

**Figure 4:**
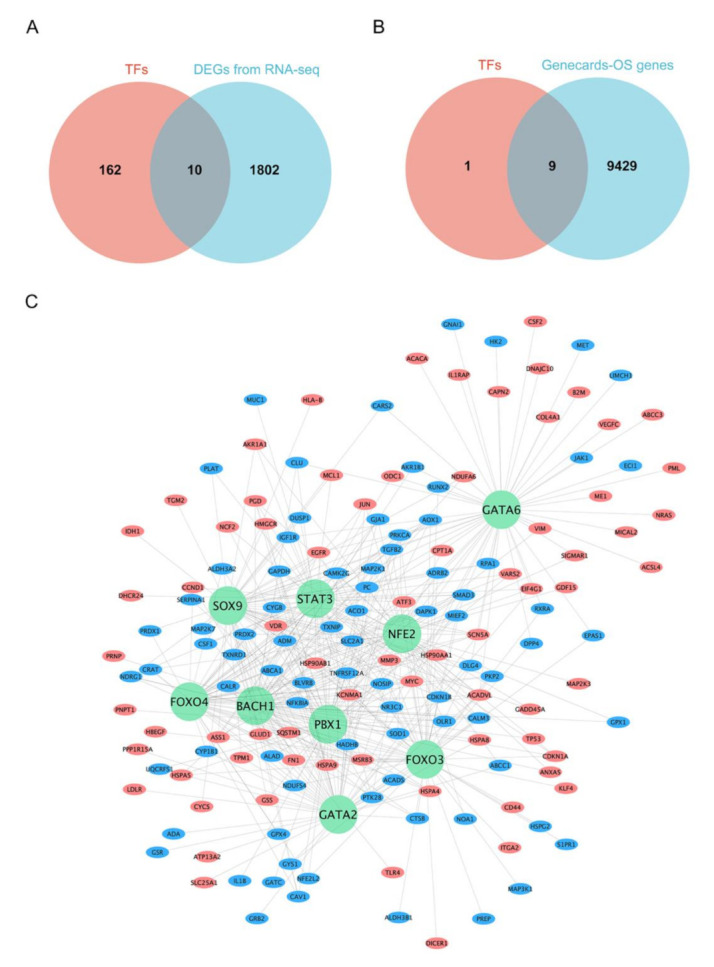
Prediction of TFs for DEGs and construction of oxidative stress-associated TF-mRNA regulatory network. (**A**) Ten TFs with altered expression after PS-NP exposure in BEAS-2B cells. (**B**) Nine TFs that related to oxidative stress. “OS” represents oxidative stress. (**C**) Oxidative stress-associated TF-mRNA regulatory network of BEAS-2B cells in response to PS-NP exposure. The green circles represent TFs related to oxidative stress. The red ellipses represent up-regulated DEGs related to oxidative stress while blue ellipses represent down-regulated DEGs related to oxidative stress.

Multiple biological processes were regulated by TFs through binding to promoter regions, resulting in the transcriptional activation of target genes. Therefore, the prediction of the TFs could help to reveal the internal mechanism of DEGs. The DAVID database was used to predict the key TFs that regulated DEGs. From our analysis, a total of 172 TFs were enriched with a *p*-value < 0.05, of which only ten TFs were differentially expressed after PS-NP exposure (Figure 4A).

In order to deeply explore the mechanism of oxidative stress triggered by PS-NPs at the molecular level, the GeneCards database was used to collect the genes related to oxidative stress and a total of 9438 genes were obtained. We intersected ten TFs with 9438 oxidative stress-associated genes. Interestingly, Venn analysis showed that nine of the ten TFs overlapped, suggesting that the nine genes may be the key TFs regulating oxidative stress induced by PS-NPs (Figure 4B). Furthermore, we intersected the 9438 genes with the DEGs that were regulated by the nine TFs (Supplementary Figure S2A–I) and constructed the oxidative stress-associated TF-mRNA regulatory network (Figure 4C).

### 3.5. Combined Analysis with Rat Lung Injury Models

**Figure 5:**
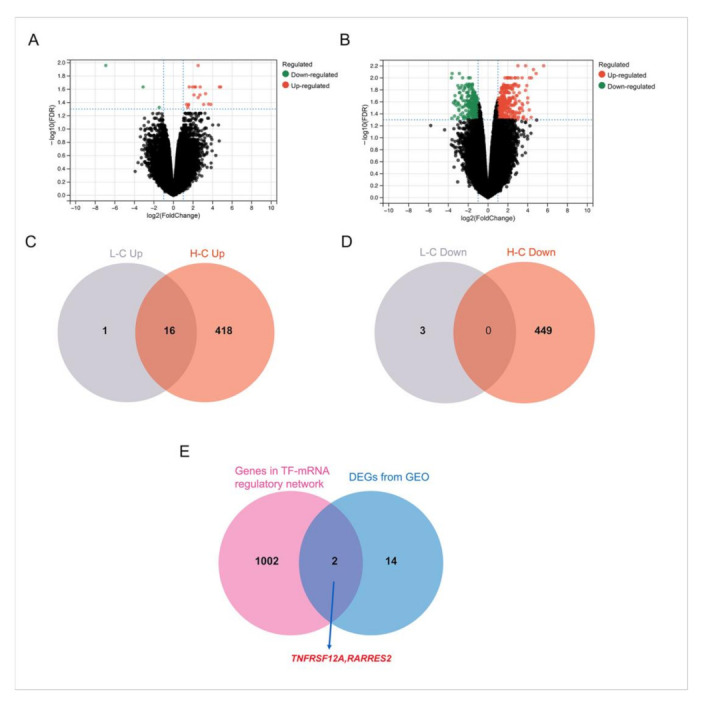
Combined analysis with rat lung injury models. (**A**,**B**): Volcano plots of significantly changed genes in rat lung tissues with acute pulmonary embolism induced by polystyrene micro-spheres of diverse concentrations. (**A**) low-dose group vs control group and (**B**) high-dose group vs control group). (**C**,**D**): Up-regulated genes (**C**) and down-regulated genes (**D**) with consistent trend in two concentrations of polystyrene treated groups. “L-C” denotes low-dose group vs control group, and “H-C” denotes high-dose group vs control group. (**E**) overlap of DEGs from our RNA-seq and GEO data.

Due to the ethical problems of obtaining human tissues and the fact that in vitro experiments cannot fully simulate the microenvironment in vivo, evidence from animal models is a good supplement for our research. Gene expression profiles of lung tissue from rats, which were exposed to polystyrene microspheres and “vehicle” control, were obtained from the GEO dataset and analyzed by GEO2R. Compared with the control groups, 20 genes were significantly altered in the low-dose group, with 17 genes up-regulated and 3 genes down-regulated (Figure 5A). Moreover, the high-dose group witnessed 883 genes change, of which 434 genes were up-regulated and 449 genes were down-regulated (Figure 5B). Notably, 16 genes showed a consistent trend in both treatment groups relative to the control group, which were all up-regulated (Figure 5C,D). Venn analysis showed that 2 genes (TNFRSF12A, RARRES2) overlapped between the genes from TF-mRNA regulatory network and the DEGs in the GEO data (Figure 5E). TNFRSF12A was up-regulated in both data while RARRES2 was down-regulated in our RNA-seq data but up-regulated in the GEO data. Interestingly, TNFRSF12A has been reported many times in previous studies to engage in oxidative stress, and evidence shows that TNFRSF12A plays an important role in respiratory diseases [28,29]. Thus, more effort will be made to investigate whether TNFRSF12A contributes to PS-NP-induced lung injuries through oxidative stress in our future studies.

### 3.6. Gene Expression Validation by qRT-PCR

**Figure 6:**
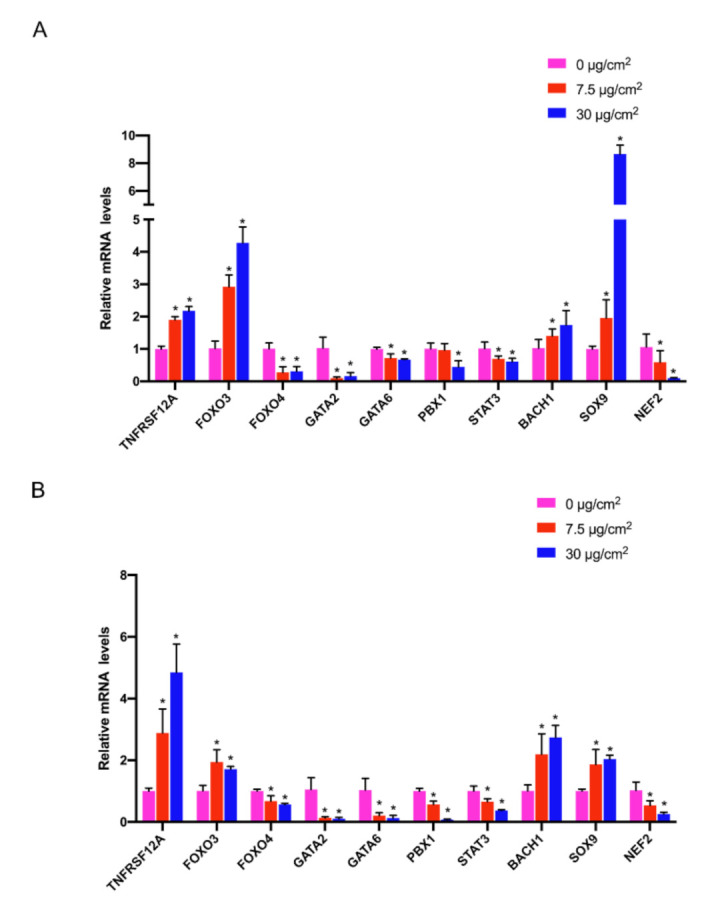
Gene expression validation by qRT-PCR. (**A**,**B**): Gene expression of BEAS-2B cells (**A**) and HPAEpiC cells (**B**) after exposure to different concentrations of PS-NPs through qRT-PCR validation. * *p*-value <  0.05 indicates statistically significant differences from the control.

Based on the above results, nine pivotal TFs and TNFRSF12A were selected for qRT-PCR validation, including four up-regulated genes (BACH1, SOX9, FOXO3, TNFRSF12A) and six down-regulated genes (GATA2, GATA6, STAT3, PBX1, NFE2, FOXO4). BEAS-2B cells and HPAEpiC cells were treated with PS-NPs with concentrations of 7.5 μg/cm^2^ and 30 μg/cm^2^ for 24 h according to our previous study [27]. Fortunately, expression of the ten genes showed a consistent trend with that in RNA-seq in both BEAS-2B cells (Figure 6A) and HPAEpiC cells (Figure 6B).

## 4. Discussion

Nanoplastics have become predominant contaminants nowadays, which has raised great concern about their toxicity [30]. Due to the presence of nanoplastics in the air, they are inevitably inhaled and enriched in the respiratory system. As a result, there is a pressing need to research lung injury and the underlying mechanisms caused by nanoplastics. In this context, we performed transcriptomic analysis to gain a comprehensive understanding of related genes and potential pathways which may be engaged in the cytotoxicity of BEAS-2B cells induced by PS-NP exposure. Further, we obtained the key TFs regulating DEGs and constructed an oxidative stress-associated TF-mRNA regulatory network. Next, we combined animal experiments data from the GEO dataset with our RNA-seq data for joint analysis and screened out the core gene. Finally, we verified these pivotal genes in BEAS-2B cells and HPAEpiC cells using qRT-PCR. By utilizing these approaches, we aimed to provide new insight into exploring the inhalation toxicity of nanoplastics.

Here we had shown that PS-NPs lead to dose-dependent cytotoxicity in BEAS-2B cells. In order to more accurately reveal the dynamic changes of genes in BEAS-2B cells induced by PS-NP exposure, we chose three groups (control group, low-dose group and high-dose group) for transcriptome sequencing. A total of 1812 genes were changed with a consistent trend in the two treated groups. Notably, approximately 700 genes had significant changes in low- but not high-dose-treated cells, and these genes may play crucial roles at sublethal stages. Sublethal damage caused by low-concentration treatments of either the PS-NPs in this study or other irritants is often reversible, and after the elimination of the irritant environment, the cells may be able to return to their normal state. Therefore, studying the genes that change in the low-concentration group alone could also guide the investigation of the potential repair mechanism of the injury, which represents a significant step forward in the prevention and treatment of diseases. Our present study mainly focused on those pivotal genes that play a role in both the onset and late stages of lung injuries, so we selected the intersection genes of the low-dose group and the high-dose group, which were then used for GO analysis and KEGG pathway analysis. According to GO analysis, approximately 1/4 of the GO terms exhibited an association with oxidative stress, such as oxidation-reduction process, oxidoreduction coenzyme metabolic process, oxidoreductase activity and so on. Interestingly, we also found clear links between KEGG pathways and oxidative stress. For example, ferroptosis, as a unique cell death mechanism, was caused by unrestricted lipid peroxidation [31,32]. It was found that the disturbance of the oxidative balance and the generation of oxidative stress were the main features of ferroptosis [33]. Moreover, some other enriched KEGG pathways, such as PI3K-Akt and the HIF-1 signaling pathways were also reported to be involved in the regulation of oxidative stress [24,25,26,27,28,29,30,31,32,33,34,35,36,37]. On the basis of the above information, the DCFH-DA staining assay, the most commonly used probe to detect intracellular H_2_O_2_ and oxidative stress, was used in this study to verify the results of the KEGG and GO analyses. We found that ROS levels remarkably increased in BEAS-2B and HPAEpiC cells after PS-NP exposure for 24 h. Since previous evidence showed that oxidative stress induced by the excessive accumulation of ROS acted as the critical adverse key event to initiate downstream signaling cascades and ultimately caused cytotoxicity [38], it could be argued that PS-NPs may contribute to the loss of viability in lung cells by activating oxidative stress.

As one type of DNA-binding protein, TFs are considered the control panel of the genome. TFs play the important role in transcriptional regulation and directly shape organism phenotypes [39]. In the current study, we identified the ten key TFs regulating DEGs using the DAVID database. The functional annotation analysis and DCFH-DA results hinted that PS-NP treatment induced significant oxidative stress in lung cells. Therefore, we supposed that these key TFs were most likely related to oxidative stress. Consistent with our conjecture, nine of the ten TFs were found to be associated with oxidative stress and the ranking is FOXO3 > STAT3 > FOXO4 > BACH1 > SOX9 > GATA2 > GATA6 > NFE2 > PBX1 according to the relevance score based on the Genecards database. FOXO3, which got the highest relevance score among the nine genes, was proved to act as a central integration hub for various biological processes [40], especially for oxidative stress. Aucello et al. [41] reported that the localized accumulation of oxidative stress caused muscle atrophy through the activation of an autophagic pathway which was mediated by FOXO3. In addition, FOXO3 was found to be required for the regulation of oxidative stress in erythropoiesis [42]. To a certain extent, these studies confirmed the credibility of the nine oxidative-stress-associated TFs we screened. The above information suggested that oxidative stress may play a key role in PS-NP-induced lung injuries. Therefore, we further constructed a regulatory network using all oxidative stress-associated DEGs and the nine TFs to visualize their interaction. Since each TF regulated a very large number of DEGs, we only selected the top 30 genes for each TF to show in the regulatory network.

Considering the process of lung injury caused by nanoplastics is dynamic and complex, it is of great necessity to add multiple experimental models for comprehensive analysis. Besides the DEGs retrieved from our experiments in vitro, we incorporated the rat lung injury models induced by polystyrene microspheres from the GEO database into this current study. We obtained 16 candidate genes from the microarray analysis of rat lung tissues. After intersecting these findings with the genes of the TF-mRNA regulatory network, we finally identified TNFRSF12A (also known as Fn14, TWEAKR, CD266) as the pivotal gene with a consistent trend in our RNA-seq data and the GEO data. Of note, Fn14, the receptor for the Tumor necrosis factor (TNF) superfamily cytokine TWEAK, is well-known for its important role in diverse pathologic processes including oxidative stress, inflammation, angiogenesis, carcinogenesis, proliferation and death [43,44,45,46]. Accumulating evidence has shown that Fn14 is involved in oxidative stress since it combines with TWEAK. According to Madrigal-Matute et al. [28], the TWEAK/Fn14 interaction increased oxidative stress via activating NADPH oxidase in macrophages. From their investigation, the TWEAK-induced ROS production was abrogated after the genetic silencing of Fn14 in the murine macrophage cell line. Consistent with the results in vitro, the deletion of Fn14 in an APOE-/- background murine resulted in a 50% reduction in the proportion of macrophages that were DHE and 8-hydroxydeoxyguanosine positive. Furthermore, it was reported that TWEAK/Fn14 promoted oxidative stress in endothelial cells through the AMPK/PGC-1α/MnSOD signaling pathway [29]. Recently, several pieces of evidence also suggested that Fn14 was engaged in the development of respiratory diseases [47]. Compared with normal lung tissue, Fn14 expression in Pulmonary Microvascular Endothelial Cells (PΜVECs) was significantly increased in mice with septic acute lung injury (ALI). Moreover, an Fn14 blockade on PΜVECs improved the outcome of sepsis-induced acute lung injury [48]. Furthermore, TWEAK induced the production of IL-8 and GM-CSF in BEAS-2B cells via Fn14. In view of the fact that IL-8 and GM-CSF were considered the typical inflammation-related cytokines [49], it suggested that interaction between TWEAK and Fn14 may promote inflammatory responses in the airways. Regrettably, we did not find any more common DEGs between the GEO dataset and our RNA-seq data due to the species difference between humans and rats, and the fact that the modeling methods were not completely equivalent. With that said, at the very least, our study offered the hypothesis that Fn14 would play a pivotal role in nanoplastic-induced lung injuries via activating oxidative stress. To avoid the possible false positive error and technological error, qRT-PCR was carried out to verify the RNA-seq consequences in two types of normal human lung epithelial cells. Notably, all the genes, including the nine TFs and FN14, showed a consistent trend with the data from the RNA-seq, further implying that results from RNA-seq were credible, and more experiments on these genes should be conducted in the future to elucidate its concrete mechanism.

Previous studies on the respiratory toxicity of nanoplastics are far from enough. In this study, we made the following efforts to fill the gaps in this research field. Firstly, instead of a single exposure concentration, we selected different treatment concentrations of PS-NPs in lung cells for RNA-seq simultaneously, allowing us to identify the altered genes with a consistent trend. Secondly, we comprehensively analyzed the underlying mechanisms, and the elevation of ROS was validated with fluorescence. Thirdly, the results of animal experimental data were also included to make our study more reliable. Future studies will employ oxidative stress inhibitors and animal models of lung injury caused by respiratory exposure to nanoplastics to establish the involvement of oxidative stress as a critical molecular event, laying the groundwork for further investigation into nanoplastics-induced inhalation toxicity.

## 5. Conclusions

Overall, our study revealed the potential respiratory toxicity of nanoplastics. We confirmed that nanoplastics indeed inhibited the viability of human lung epithelial cells mediated by oxidative stress. Through RNA-seq and bioinformatics analysis, we screened nine key TFs that regulated nanoplastic-induced oxidative stress in BEAS-2B cells and constructed the regulatory network. Combined with a GEO database analysis, we identified that FN14, a star molecule involved in the regulation of oxidative stress, may act as an intriguing target in lung injuries caused by nanoplastics.

## Data Availability

All data reported in this study are available from the corresponding author upon any reasonable request.

## Supplementary Materials

The following supporting information can be downloaded at: https://www.mdpi.com/article/10.3390/nano12193507/s1, Figure S1: Heatmaps of DEGs in BEAS-2B cells after PS-NPs exposure of diverse concentrations compared with the control group; Figure S2: The DEGs related to oxidative stress that regulated by 9 TFs; Table S1: The total of 9438 oxidative stress-related candidate genes that enriched in GeneCards database. Table S2: The primer sequences for qPCR.
