# Supplementary material for: Polystyrene Nanoplastics Induce Lung Injury via Activating Oxidative Stress: Molecular Insights from Bioinformatics Analysis
Source: Nanomaterials (Basel). 2022 Oct 7;12(19):3507. doi: 10.3390/nano12193507 (PMC9565894; doi:10.3390/nano12193507)
Supplement: Supplementary file 1 [file nanomaterials-12-03507-s001.zip › nanomaterials-1940326 - Supplementary Materials.pdf]

nanomaterials

MDPI

Supplementary Materials

Polystyrene Nanoplastics Induce Lung Injury via Activating Oxidative Stress: Molecular Insights from Bioinformatics Analysis

Tianyi Zhang, Sheng Yang, Yiling Ge, Xin Wan, Yuxin Zhu, Jie Li, Lihong Yin, Yuepu Pu and Geyu Liang *

Key Laboratory of Environmental Medicine Engineering, School of Public Health, Southeast University, Ministry of Education, Nanjing 210009, China
* Correspondence: lianggeyu@163.com; Tel.: +86-25-83272572; Fax: +86-25-83324322.

This PDF file includes:
Figures S1–S2 and Table S2
Table S1 was uploaded separately due to the scale of the data.

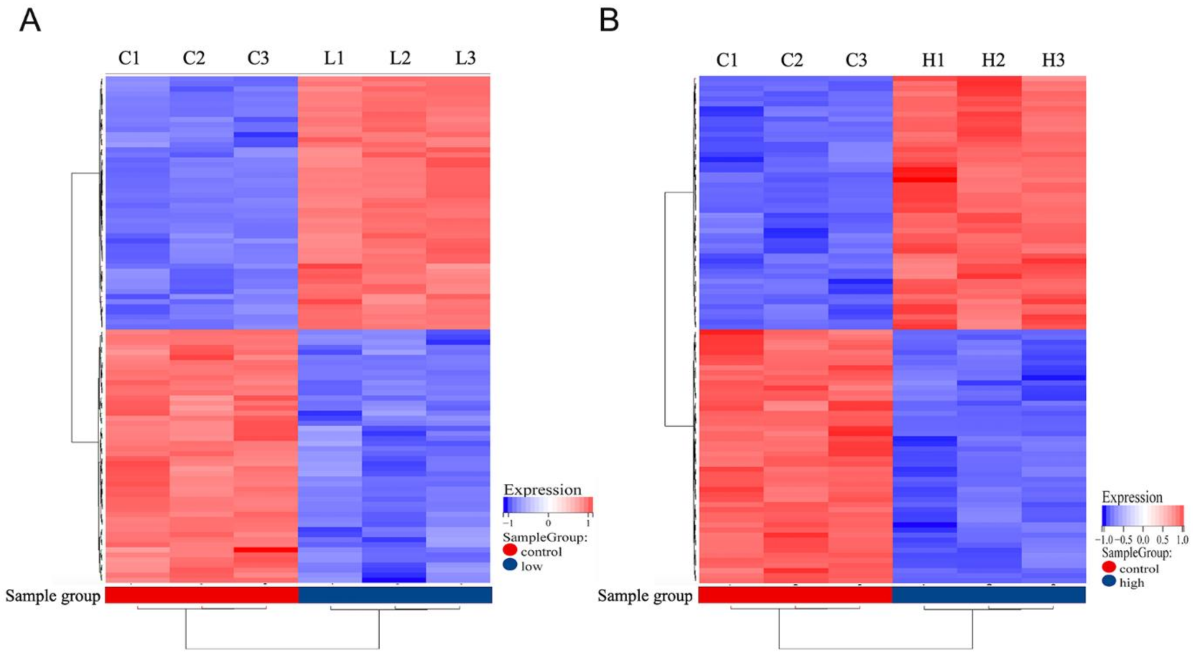

Figure S1. Heatmaps of DEGs in BEAS-2B cells after PS-NPs exposure of diverse concentrations compared with the control group. (**A**) low dose group vs control group. (**B**) high dose group vs control group.

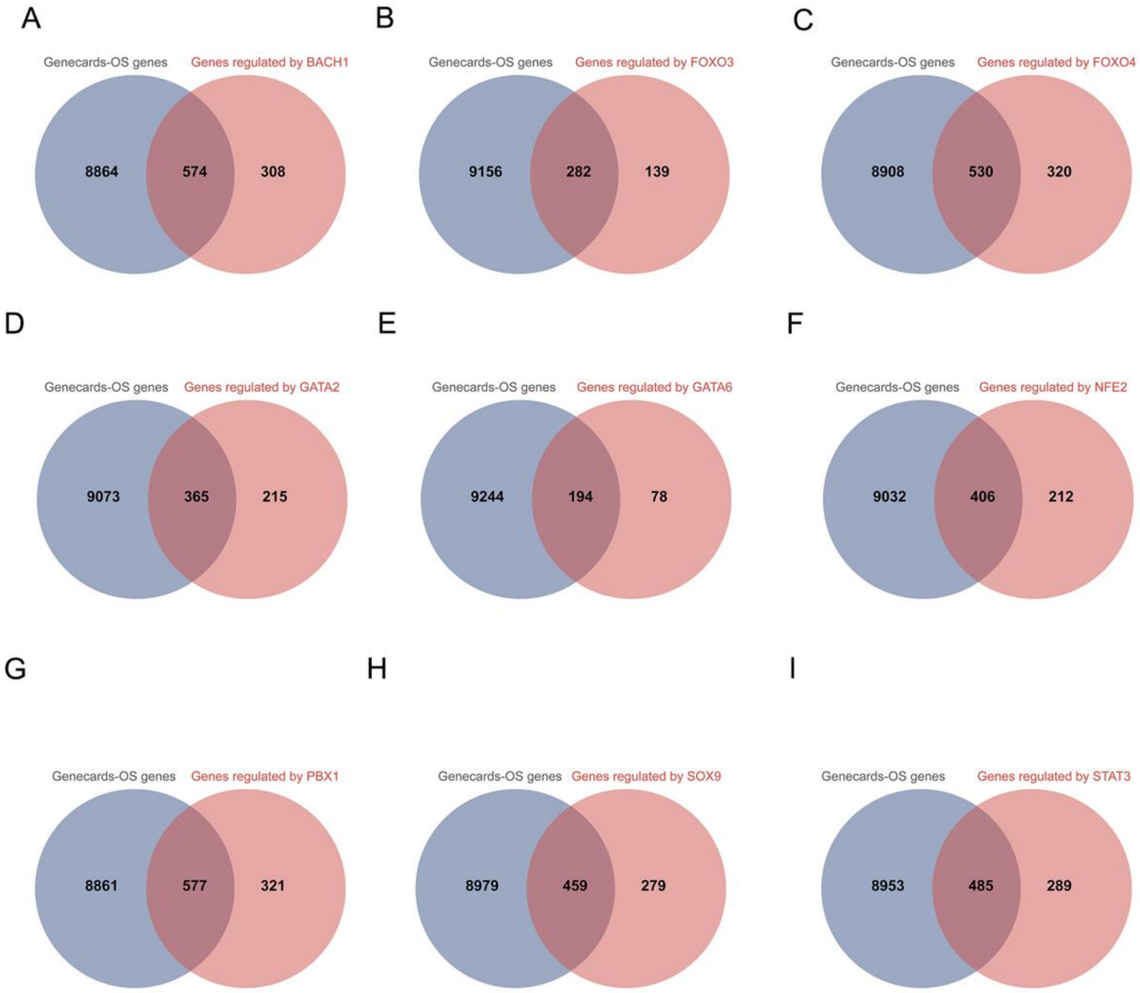

Figure S2. (**A–I**) The DEGs related to oxidative stress that regulated by BACH1 (**A**), FOXO3 (**B**), FOXO4 (**C**), GATA2 (**D**), GATA6 (**E**), NFE2 (**F**), PBX1 (**G**), SOX9 (**H**) and STAT3 (**I**). “OS” represents oxidative stress.

Table S2. The primer sequences for qPCR.

Gene Symbol	Forward Sequence (5′ to 3′)	Reverse Sequence (5′ to 3′)
Bach1	CTCAGCCTTAATGACCAGCGG	GCCTACGATTCTTGAGTGGAAG
Gata2	ACTGACGGAGAGCATGAAGAT	CCGGCACATAGGAGGGGTA
Gata6	CTCAGTTCCTACGCTTCGCAT	GTCGAGGTCAGTGAACAGCA
Sox9	AGCGAACGCACATCAAGAC	CTGTAGGCGATCTGTTGGGG
Pbx1	GACAACTCAGTGGAGCATTCA	CTCTCGCAGGAGATTCATCAC
Nfe2	CGGCGCAGCGAATATGTAGA	CCGACGTTCATCCCGACTC
Foxo3	CGGACAAACGGCTCACTCT	CGGATCGAGTTCTTCCATCCTG
Foxo4	CCGGAGAAGCGACTGACAC	CCTTCGAGGAGCAGAGTGCG
Tnfrsf12a	GGGTGCCCTCCTTCCTA	GCCAGCCCTCCTAGTGG
Gapdh	CCTGTTCCAGAGACAGCCGC	GCGCCCAATACGGCCAAATC
